# SUMOylation of synapsin Ia maintains synaptic vesicle availability and is reduced in an autism mutation

**DOI:** 10.1038/ncomms8728

**Published:** 2015-07-15

**Authors:** Leo T. -H. Tang, Tim J. Craig, Jeremy M. Henley

**Affiliations:** 1 School of Biochemistry, Medical Sciences Building, University of Bristol, University Walk, Bristol BS8 1TD, UK.

## Abstract

Synapsins are key components of the presynaptic neurotransmitter release machinery. Their main role is to cluster synaptic vesicles (SVs) to each other and anchor them to the actin cytoskeleton to establish the reserve vesicle pool, and then release them in response to appropriate membrane depolarization. Here we demonstrate that SUMOylation of synapsin Ia (SynIa) at K687 is necessary for SynIa function. Replacement of endogenous SynIa with a non-SUMOylatable mutant decreases the size of the releasable vesicle pool and impairs stimulated SV exocytosis. SUMOylation enhances SynIa association with SVs to promote the efficient reclustering of SynIa following neuronal stimulation and maintain its presynaptic localization. The A548T mutation in SynIa is strongly associated with autism and epilepsy and we show that it leads to defective SynIa SUMOylation. These results identify SUMOylation as a fundamental regulator of SynIa function and reveal a novel link between reduced SUMOylation of SynIa and neurological disorders.

## Abstract (web summary)

Synapsins anchor synaptic vesicles (SVs) to the actin cytoskeleton to establish the reserve vesicle pool. Here Tang *et al*. show that SUMOylation of synapsin 1a enhances its interaction with SVs to promote efficient reclustering following stimulation, and a mutation linked to autism and epilepsy leads to defective SUMOylation.

## Graphical abstract

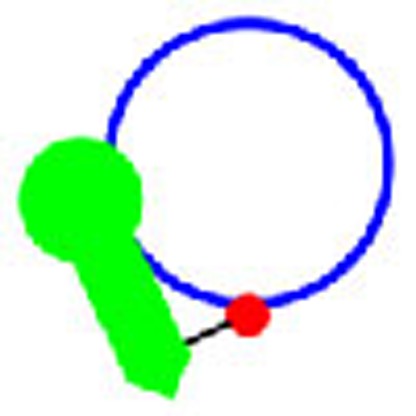

The coordinated release of neurotransmitter from synaptic vesicles (SVs) is fundamental to synaptic function and plasticity. It is generally accepted that SVs are organized into distinct pools at the presynapse including the readily releasable pool that is synchronously released immediately following stimulation and the reserve pool (RP), that is mobilized during prolonged stimulations. Vesicles that are refractory to release are often referred to as the resting pool^1^. SynIa maintains the RP of SVs by tethering them to each other and to the presynaptic actin cytoskeleton near to presynaptic release sites. Presynaptic depolarization causes SynIa phosphorylation through the CamKII and PKA pathways, which decreases binding affinity and releases the tethered SVs allowing them to move to the active zone^2^,^3^,^4^. On the other hand, phosphorylation by other kinases such as c-Src and cdk5 alters the distribution of SVs between different pools^5^,^6^.

SUMOylation is the covalent attachment of the small ubiquitin-like modifier (SUMO) to lysine residues on target proteins. In neurons, SUMOylation a key regulator of transcription and extranuclear SUMOylation plays fundamentally important roles in synaptic function^7^,^8^,^9^,^10^ and neuroprotective responses to severe stressors^11^. Recently, we reported that SUMOylation of RIM1α is required for normal SV exocytosis, demonstrating that SUMOylation also has a role in presynaptic regulation^12^.

Here we show that SUMOylation of SynIa at K687 enhances binding to SVs and facilitates synapsin-mediated SV clustering and anchoring. Replacement of endogenous SynIa with a non-SUMOylatable K687R mutant in neurons reduces the number of releasable SVs and impairs exocytosis. Furthermore, we demonstrate that an A548T mutation in SynIa, which has been linked to autism spectrum disorder (ASD) and epilepsy, reduces SynIa SUMOylation and mirrors the functional defect of non-SUMOyatable SynIa, suggesting a possible causal link between dysfunctional SynIa SUMOylation and neurological disorders.

## Results and Discussion

### Synapsin Ia is a SUMO substrate

**Figure 1:**
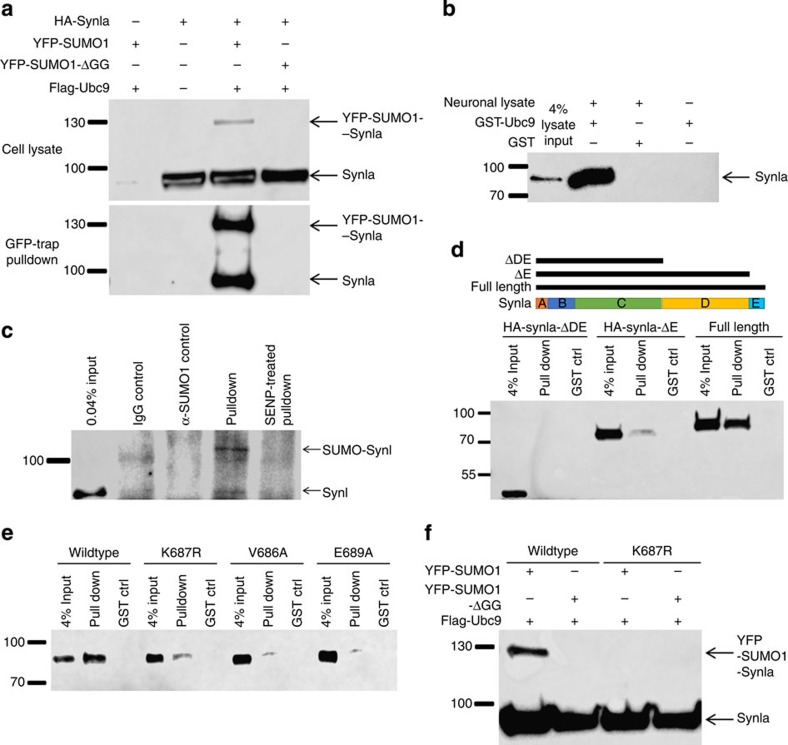
SynIa is SUMOylated at K687R. All experiments were performed at least three times with consistent results. (**a**) Representative anti-HA immunoblot showing that SynIa is robustly SUMOylated. HA-SynIa, Flag-Ubc9 and YFP-SUMO-1 were expressed in N2A cells and the lysate then subjected to GFP-trap pull down to purify YFP-SUMOylated proteins. (**b**) GST-pull-down assays showing that endogenous SynI from rat cortical neurons binds Ubc9. (**c**) Immunoprecipitation of endogenous SUMOylated SynIa from rat brain. Whole rat brain was lysed under denaturing conditions and subjected to IP with anti-SUMO-1 antibody, then immunoblotted with anti-SynI antibody. IgG Control: mouse IgG was used instead of anti-SUMO-1; α-SUMO-1 control: RIPA buffer was used instead of brain lysate. SENP-treated pull down: before the pull down, the lysate was supplemented with 25 nM SENP (catalytic domain fragment) and incubated for 1 h at room temperature. (**d**) Full-length HA-SynIa and truncation constructs lacking the E domain (ΔE) or both the D and E domains (ΔDE) were expressed in HEK293T cells and the lysates subjected to GST-Ubc9 pull down. (**e**) Point mutations of residues in the SUMO consensus site in SynIa prevent pull down by GST-Ubc9 in HEK293T cells. (**f**) K687 is the only SUMOylation site in SynIa. HA-SynIa WT or HA-SynIa K687R were expressed with Flag-Ubc9 and YFP-SUMO-1 in N2A cells and lysates were immunoblotted with anti-HA antibody.

To validate SynIa as a SUMO-1 substrate, we used a SUMOylation assay system in which we exogenously expressed HA-SynIa, Flag-Ubc9 and either YFP-SUMO or non-conjugatable YFP-SUMO-ΔGG in N2A cells. A SUMOylated SynIa band was detected in the cells expressing YFP-SUMO but not YFP-SUMO-ΔGG (Fig. 1a, upper panel). This band was also present in green fluorescent protein (GFP)-trap pull downs of YFP-SUMO from the N2A lysate, further confirming it corresponds to SUMOylated SynIa (Fig. 1a, lower panel). We attribute the presence of an equal density unmodified SynIa band to dimerization of SynIa and SynIa-SUMO.

Ubc9 binding to the target protein is a critical step in SUMOylation. Consistent with SynIa being a robust SUMO substrate, endogenous SynIa in cultured cortical neuronal lysates is strongly retained by GST-Ubc9 pull downs (Fig. 1b).

Importantly, we also detected endogenous SUMOylated SynIa in neurons by anti-SUMO co-immunoprecipitation from whole-brain lysate. As expected, anti-SynI antibody immunoblots of the immunoprecipitated protein revealed a ∼110-kDa band corresponding to SUMOylated SynIa. Critically, this band was removed by treatment with the deSUMOylating enzyme SENP1 (Fig. 1c). Again, we attribute the presence of unmodified SynIa in this co-immunoprecipitation to dimerization.

SynIa is a multidomain protein; therefore, to define the SUMOylation site(s), we first generated and expressed SynIa mutants lacking the E domain (ΔE) or both D and E domains (ΔDE) in HEK293T cells and performed GST-Ubc9 pull-down assays (Fig. 1d). Removal of the E domain of SynIa greatly decreased the interaction with GST-Ubc9, and additional deletion of the D domain completely abolished the interaction (Fig. 1d). These data indicate that Ubc9 binds mainly to a region within the E domain, but may also have some interaction within the D domain.

Consistent with this, the E domain contains the SUMO consensus sequence VKAE at residues 686–689. Mutation of the Val686 or Glu689 residues flanking the target lysine to Ala disrupted the SynIa—GST-Ubc9 interaction (Fig. 1e) and mutating Lys687 to Arg completely prevented SynIa SUMOylation (Fig. 1f).

The E domain is involved in dimerization, vesicle clustering, presynaptic targeting, cytoskeleton interaction and vesicle release^13^,^14^. All synapsin a-isoforms (SynIa, SynIIa and SynIIIa) contain highly conserved E domains^15^,^16^ and SynIIa could also be SUMOylated in N2A cells, even though the consensus sequence is AKAE in this protein (Supplementary Fig. 1a). As shown in Fig. 1e, V686A inhibited Ubc9 binding to SynIa in HEK293T cells. However, in N2A cells, this mutation could still be SUMOylated (Supplementary Fig. 1b), indicating that an additional factor present in this neuronal cell line, presumably an E3 ligase, is required to enhance SUMOylation. Sequence alignment shows that K687 is also conserved across a-isoforms of synapsins in vertebrates (Supplementary Fig. 1c). However, despite its importance, the molecular mechanisms underlying the function of the E domain is not well characterized.

### Preventing SynIa SUMOylation causes defective SV exocytosis

The SUMO consensus sequence VKAE is present in SynIa across most vertebrate species consistent with SUMOylation playing an important role in SynIa function throughout evolution. We therefore tested the effect of acutely reducing endogenous SynIa by short hairpin RNA (shRNA) knockdown and replacement with shRNA-insensitive wild-type SynIa or the non-SUMOylatable mutant. Transfection of hippocampal neurons with SynI shRNA decreased in SynI levels to 50% of control levels. Transfection with shRNA-resistant wild-type or K687R SynIa constructs restored SynI to near-endogenous levels (Supplementary Fig. 2).

**Figure 2:**
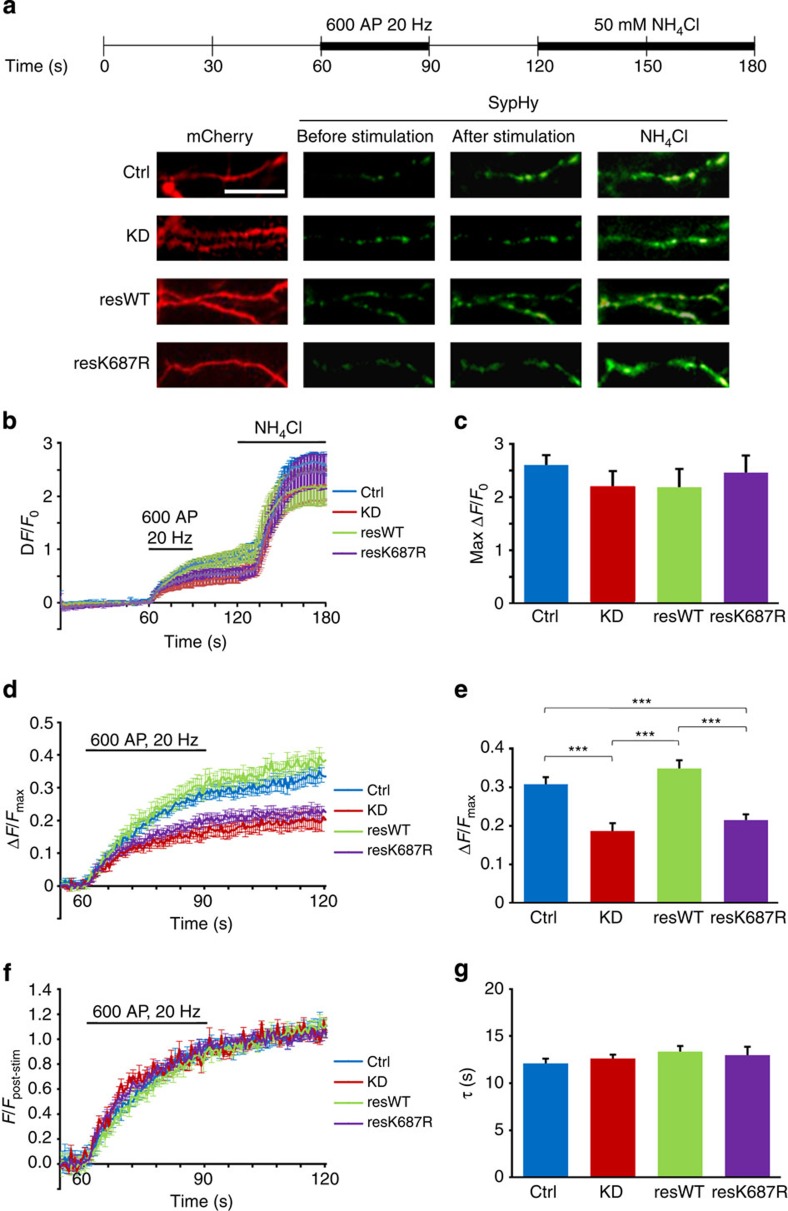
SUMOylation of SynIa regulates SV exocytosis. (**a**) SypHy assay on SynIa knockdown and molecular replacement in DIV 15 hippocampal neurons, transfected and imaged live 5 days later. The bicistronic expression constructs incorporate mCherry to indicate transfection. The schematic shows the experimental time course and the images are representative of the SypHy signal before and after stimulation (*t*=0 and 90 s, respectively), and after NH_4_Cl perfusion (*t*=180 s). Control (Ctrl, *n*=13), Knockdown (KD, *n*=12), KD-rescue WT (resWT, *n*=12) and KD-rescue K687R (resK687R, *n*=12). Scale bar, 10 μm. (**b**) Average traces of Δ*F*/*F*_0_ over the whole course of assay, plotted as mean±s.e.m. (**c**) Quantification of data shown in **b** plotted as the mean±s.e.m. of the average Δ*F*_max_ (*t*=175–180 s)/*F*_0_, providing a measure of the total SV pool in the presynapse. One-way analysis of variance (ANOVA) showed no significant difference between any values. *n* numbers as in **a**. (**d**) Replacement of endogenous SynIa with SynIa K687R causes defective exocytosis. SypHy fluorescence data were normalized to the maximum SypHy signal obtained after NH_4_Cl application (Δ*F*/*F*_max_) and plotted as the mean±s.e.m. *n* numbers as in **a**. (**e**) Quantification of data shown in **d** plotted as the mean±s.e.m. of the average Δ*F*/*F*_max_ *t*=90–100 s, providing a measure of relative overall SV exocytosis against total SV pool size. ***P<0.001, one-way ANOVA, *post hoc* test with Bonferroni correction. (**f**) Replacement of endogenous SynIa with SynIa K687R has no effect on the kinetics of SV release. Change in SypHy signal normalized against average signal after stimulation (Δ*F*/*F*_post-stim_), plotted as mean±s.e.m. against time. *n* numbers as in **a**. (**g**) Δ*F*/*F*_post-stim_ between 60 and 120 s were fitted to exponential curves and the time constant *τ* plotted as mean±s.e.m. Significance tested with one-way ANOVA, *post hoc* test with Bonferroni correction.

We investigated the dynamics of SV exocytosis using the genetically encoded reporter Synaptophysin-pHluorin (SypHy)^17^. Hippocampal neurons transfected with both SypHy and knockdown-rescue constructs were subjected to 600 action potentials at 20 Hz using electrical field stimulation to evoke SV exocytosis of all releasable vesicles (that is, readily releasable pool and RP). These experiments were performed in the presence of the proton pump inhibitor bafilomycin1A to prevent reacidification of endocytosed SVs so that each SV is counted only once on first-time release^18^. The SypHy fluorescence was expressed as the change in fluorescence normalized against the basal signal (Δ*F*/*F*_0_), presented in Fig. 2a,b. To assess the average size of the SV pool in the presynapse, the mean level of Δ*F*/*F*_0_ at the end of the experiment (during 50 mM NH_4_Cl, *F*_max_) was calculated and is presented in Fig. 2c. No significant difference was observed between any conditions, indicating that the knockdown of Syn1 or replacement with a non-SUMOylatable mutant did not change the total SV pool. To selectively assess the size and dynamics of the releasable pool, we normalized the change in fluorescence against *F*_max_ (Δ*F*/*F*_max_). Consistent with previous reports using knockout mice, acute shRNA-mediated knockdown of SynI significantly decreased the total level of SV exocytosis, measured by the average Δ*F*/*F*_max_ after stimulation, indicating a reduction in releasable vesicle pool size^19^,^20^,^21^. Importantly, a scrambled shRNA control had no effect (Supplementary Fig. 3a,b). Replacement of the knockdown with wild-type SynIa returned SV exocytosis to control levels. However, replacement with the SynI-K687R mutant did not rescue the normal release profile with levels of exocytosis indistinguishable from the knockdown neurons (Fig. 2d,e). This result cannot be attributed to a dominant-negative effect of K867R Syn1a, as expression of the K687R mutant in non-knockdown neurons did not cause any effect on SV exocytosis (Supplementary Fig. 3c,d). These data demonstrate that SynIa SUMOylation is required for normal levels of SV exocytosis.

This decrease in SV exocytosis could be mediated by reduced vesicle fusion or by a decrease in the number of releasable vesicles. We therefore assessed the rate of exocytosis by measuring the time constant of a single exponential growth curve fit to Δ*F*/*F*_post-stim_. As shown in Fig. 2f,g, there was no difference in the rates of vesicle fusion in neurons in which SynIa was knocked down, or knocked down and replaced with either wild-type SynIa or with the K687R mutant. Therefore, we attribute the decreased exocytosis in neurons expressing the non-SUMOylatable SynIa-K687R to a reduction in the size of the releasable vesicle pool, that is, fewer SVs available for release, rather than a defect in vesicle exocytosis.

We also tested if SV exocytosis is affected by a global decrease in SUMOylation by overexpressing the catalytic domain of SENP. Consistent with our results using non-SUMOylatable SynIa, overexpression of constitutively active SENP induced a decrease in the overall amount of SV exocytosis, whereas overexpression of the protease inactive mutant SENP C603S had no effect (Supplementary Fig. 4). This decrease was considerably more pronounced than molecular replacement with SynIa-K687R, suggesting that other proteins involved in SV regulation are also SUMO targets. While we cannot definitively exclude the possibility that downstream effects induced by decreased SUMOylation of non-synaptic proteins, for example, transcription factors, could contribute to this effect, our data are consistent with previous studies that identify other presynaptic SUMO substrates^12^,^22^,^23^.

### SUMOylation of SynIa enhances binding to SVs

**Figure 3:**
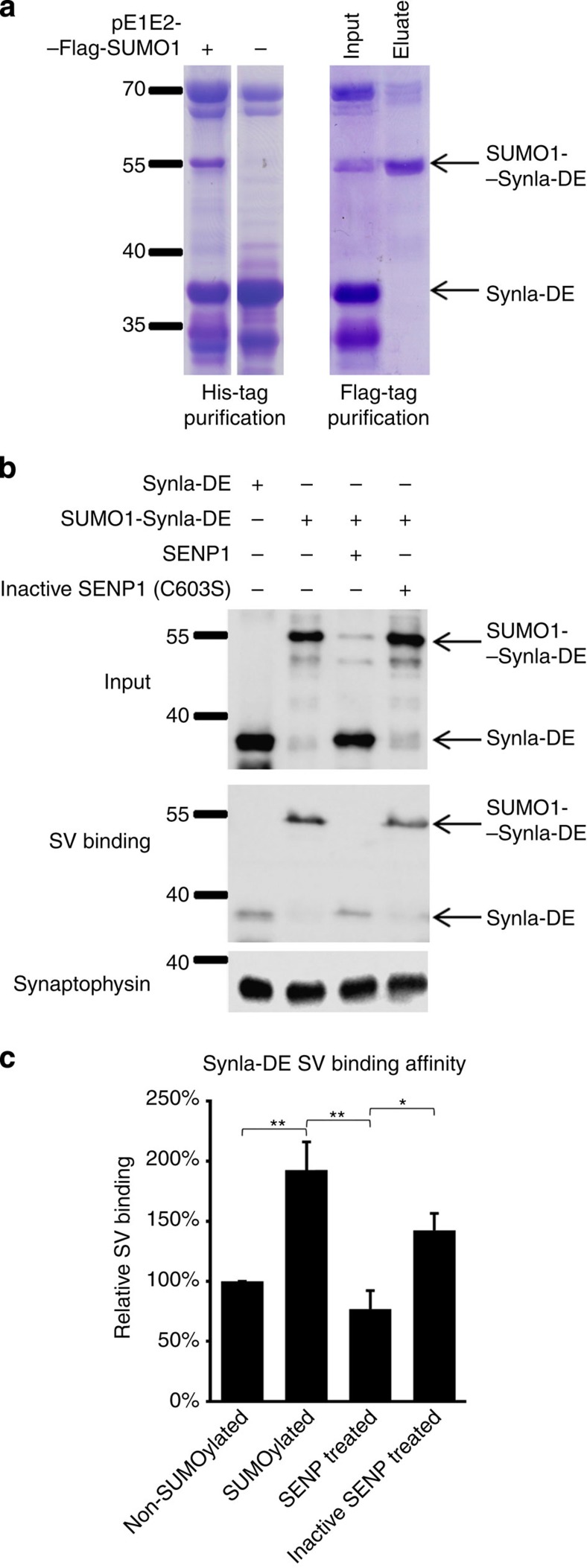
SUMOylation of SynIa promotes SV binding. (**a**) Preparation of SUMOylated SynIaDE fragment. HA-His-SynIaDE was bacterially expressed with or without co-expression of the E1/E2/Flag-SUMO construct^44^. Eluates containing SUMOylated SynIaDE were purified using an anti-Flag antibody resin and eluted with Flag peptide. Representative Coomassie-stained SDS–polyacrylamide gel electrophoresis (SDS–PAGE) of the input and eluate from both purification steps are shown. (**b**) SVs were extracted from rat brain and mixed with purified recombinant HA-His-SynIaDE (*n*=6) or SUMO-HA-His-SynIaDE (*n*=6) with active or inactive SENP (both *n*=4) to de-SUMOylate SynIaDE (*n*=4). The SVs were isolated by ultracentrifugation, resuspended and analysed by SDS–PAGE and immunoblotting for SynIaDE using anti-HA antibody. Anti-synaptophysin blot (below) shows equal loading of the SV fraction. (**c**) Quantification of binding of non-modified, SUMOylated, deSUMOylated and inactive SENP-treated SUMOylated SynIaDE to SVs. Data presented as mean±s.e.m. **P<0.01, *P<0.05 one-way ANOVA, *post hoc* test with Bonferroni correction. *n* numbers as above.

It is well known that changes in the binding affinity of SynIa to SVs plays a major role in SV dynamics^3^. Therefore, we investigated whether SUMOylation of Syn1a affects its binding to SVs, using an HA-His-tagged SynIa-DE domain fragment. SynIa-DE was co-expressed in bacteria with SUMOylation machinery with a Flag-tagged SUMOylation construct^24^,^25^. SUMOylated SynIa-DE was purified using metal-affinity purification followed of the SynIa followed by Flag-affinity purification (Fig. 3a). The purified, SUMOylated SynIa-DE was divided into equal aliquots, one of which was deSUMOylated with SENP1. The aliquots of SUMOylated and deSUMOylated SynIa-DE were then incubated with an enriched SV suspension from rat brain homogenate. The SVs were pelleted by ultracentrifugation and subjected to quantitative western blot analysis to determine the amount of bound SynIa-DE in SV fraction. SUMOylated SynIa-DE displayed significantly higher affinity binding to SVs than non-SUMOylated or deSUMOylated SynIa-DE (Fig. 3b,c). These results demonstrate that, opposite to CamKII phosphorylation of SynIa, SUMOylation increases the affinity of SynIa binding to SVs. Thus, these data support the hypothesis that SUMOylation of SynIa promotes SV binding and plays key roles in establishing and maintaining the reserve vesicle pool.

The generally accepted model of synapsin structure and function is that the D and E domains interact with SVs by binding to specific vesicle proteins, whereas the A and C domains directly bind to the vesicle phospholipid bilayer^14^,^26^,^27^. SUMOylation can alter substrate protein conformation^28^ and/or promote protein–protein interactions via SUMO-interacting motifs^29^. Enhanced association of SUMOylated SynIa to SVs may be mediated by (i) SUMOylation-dependent changes in the conformation of SynI, causing an increase in SV affinity and/or by (ii) SUMOylated SynIa binding to a SUMO-interacting motif-containing SV protein. Moreover, since CamKII phosphorylation promotes the dissociation of SynIa and the release of SVs from the actin cytoskeleton^30^, it is also possible that SUMOylation of SynIa could reduce or prevent CamKII phosphorylation.

### SUMOylation is crucial in SynIa targeting and clustering

**Figure 4:**
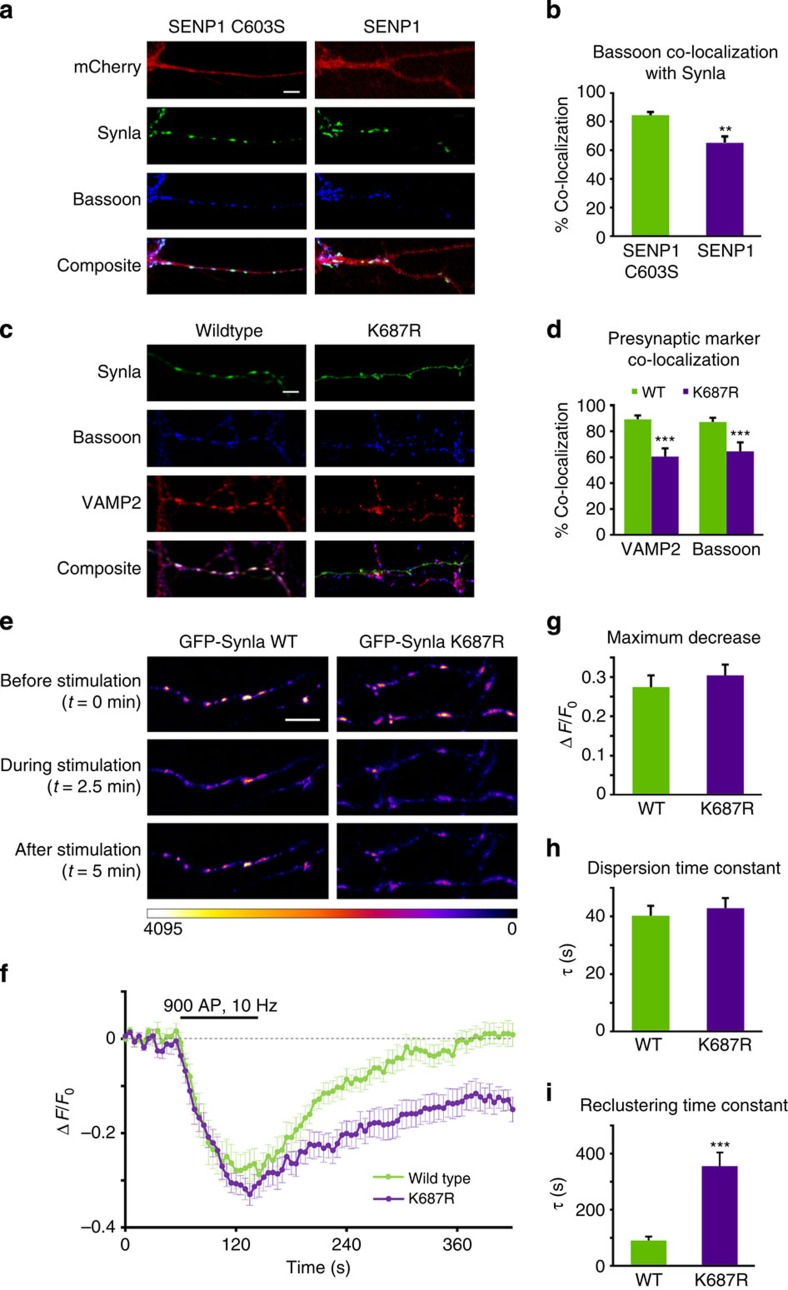
SUMOylation of SynIa modulates its presynaptic targeting and reclustering at the presynapse following stimulation. (**a**) SUMOylation is required for presynaptic targeting. WT (*n*=12) or C603S (dead mutant, *n*=12) SENP1-mCherry was overexpressed in DIV 9–10 hippocampal neurons. Cells were fixed 7 days later and immunostained with anti-SynI and anti-bassoon (presynaptic marker) antibodies. Scale bar, 5 μm. (**b**) Quantification of **a**. Co-localizing SynIa and bassoon-positive puncta were counted and divided by the total number of puncta. Data presented as mean±s.e.m. **P<0.01 against C603S mutant, unpaired Student’s *t*-test. (**c**) SUMOylation of Syn1a is required for correct presynaptic targeting. Molecular replacement of endogenous SynIa in hippocampal neurons with GFP-SynIa WT (*n*=13) or GFP-SynIa K687R (*n*=13). Neurons were transfected at DIV 9–10 and fixed and imaged 7 days later using anti-bassoon as an active zone marker, and anti-VAMP2 as SV marker. Scale bar, 5 μm. (**d**) Quantification of **c**. Synaptic co-localization of SynIa with bassoon or VAMP2 positive puncta were counted and divided by the total number of puncta. Data presented as mean±s.e.m. ***P<0.001 against wild type with respect to the same marker, unpaired Student’s *t*-test. (**e**) DIV 9–10 hippocampal neurons transfected with wild type (*n*=9) or K687R (*n*=12) GFP-tagged SynIa and imaged 7 days later. Electric field stimulation (900 AP at 10 Hz) was applied after 60 s of baseline recording and the recovery followed for 300 s afterwards. Representative images of clusters of GFP-SynIa WT and GFP-SynIa K687R before, during and after stimulation. Colour scale in arbitrary fluorescent units. Scale bar, 5 μm. (**f**) Dispersion and reclustering profiles GFP-SynIa WT and GFP-SynIa K6887R fluorescence. The data are presented as mean ± s.e.m. of the changes in punctate fluorescence (Δ*F*/*F*_0_). *n* numbers as in **e**. (**g**) Histogram showing the mean ± s.e.m. of maximum dispersion at *t*=180 s. *n* numbers as in **e**. (**h**,**i**) The dispersion (*t*=60–150 s) and reclustering (*t*=150–420 s) profiles were fitted with exponential curves and time constants presented as mean±s.e.m. ***P<0.001 against wild type, Student’s *t*-test. *n* numbers as in **e**.

Because the association of SynIa with SVs is critical for correct localization at the presynaptic terminal^31^,^32^,^33^, we next investigated the effect of SUMOylation on SynIa targeting. We overexpressed the SENP1 catalytic domain fragment in hippocampal neurons to decrease the overall cellular SUMOylation level. Endogenous SynI was then stained together with the presynaptic marker Bassoon. SynI puncta that co-localized with Bassoon were counted and normalized to the total number of SynI puncta^34^. When compared with the overexpression of a catalytically inactive mutant (C603S) of SENP1, there were significantly more SynI puncta that did not co-localize with Bassoon, suggesting that SUMOylation influences the targeting activity of SynI (Fig. 4a,b).

To ensure that the mistargetting effect was specifically due to SynIa SUMOylation, endogenous SynIa was knocked down in rat hippocampal neurons and replaced with either GFP-tagged WT or K687R SynIa, then fixed and the co-localization with bassoon and the SV marker VAMP2 assessed. As shown in Fig. 4c,d, while both WT and K687R SynIa showed punctate distributions, there was significantly lower co-localization of the K687R mutant with Bassoon or VAMP2 compared with WT SynIa. There was no difference in the intensity of VAMP2 staining caused by Syn1a K687R expression (Supplementary Fig. 5a), indicating that SVs themselves are still correctly localized at the active zone when Syn1a SUMOylation is inhibited. These results indicate that preventing SUMOylation causes a defect in presynaptic vesicle targeting of SynIa, resulting in a functional defect in the vesicle pool.

To further explore the role(s) of SUMOylation in SynIa association with SVs, we performed SynIa dispersion and reclustering assays, which measure changes in fluorescence of GFP-tagged SynI at synaptic boutons^35^. Intense synaptic activity causes SynI to diffuse away from synaptic boutons due to dissociation from the SVs. Following stimulation, SynI then reclusters at the synapse. Using this approach, we were able to determine the contribution of SUMOylation to both association and disassociation of SynIa with SVs.

We expressed GFP-tagged SynIa WT and K687R mutant in hippocampal neurons. At rest, both proteins displayed a clear punctate distribution. Electrical field stimulation of 900 AP at 10 Hz produced a significant decrease in punctate fluorescence as GFP-SynIa diffused away from synaptic boutons (Fig. 4e,f). Furthermore, WT and K687R SynIa showed a similar magnitude and time constant of dispersion (Fig. 4g,h), as well as the number of responsive puncta (Supplementary Fig. 5b). Importantly, however, the K687R mutant exhibited markedly different reclustering dynamics. Punctate fluorescence for WT SynIa completely recovered to the baseline levels 3 min after stimulation, whereas the non-SUMOylatable K687R mutant only recovered ∼50% at 3 min and had a significantly higher time constant for reclustering (Fig. 4i). These results indicate that SUMOylation does not participate in SynIa dissociation from SVs but that it is required for reclustering after synaptic activation. Furthermore, because CamKII phosphorylation mediates SynIa dissociation from SVs, these data argue against a direct interplay between phosphorylation and SUMOylation of SynIa in regulating SynIa–SV interactions. Rather, our results suggest that phosphorylation and SUMOylation control SynIa function via separately coordinated pathways.

Taken together, our data indicate that SUMOylation of SynIa promotes binding to SVs and that this is required for correct presynaptic targeting of SynIa and reclustering of SVs following stimulation. This, in turn, replenishes and maintains the reserve vesicle pool to ensure presynaptic integrity and correct dynamics of neurotransmitter release. We attribute the decrease in SV exocytosis when SynIa SUMOylation is inhibited (Fig. 2) to the defects in the establishment and maintenance of the vesicle pools, resulting in fewer vesicles being available for release during prolonged stimulation. In particular, this phenotype bears striking resemblance to that observed in sypHy exocytosis assay under cdk5 inhibition^5^, in which a shift in SV ratio between releasable and non-releasable pool was observed. One attractive possibility is that SUMOylation and cdk5 are part of a pathway that fine-tunes the ratio of releasable and non-releasable SVs.

As reported for most SUMO substrates^36^, only a small proportion of SynIa is SUMOylated at any given time. Consistent with this, our proposed mechanism of action only requires SynIa to be SUMOylated for the initiation of SV association. Once the binding is stabilized, SUMO can be removed as SynIa itself has a high affinity for SV. (Graphical representation in Supplementary Fig. 6).

### SynIa SUMOylation and disease

**Figure 5:**
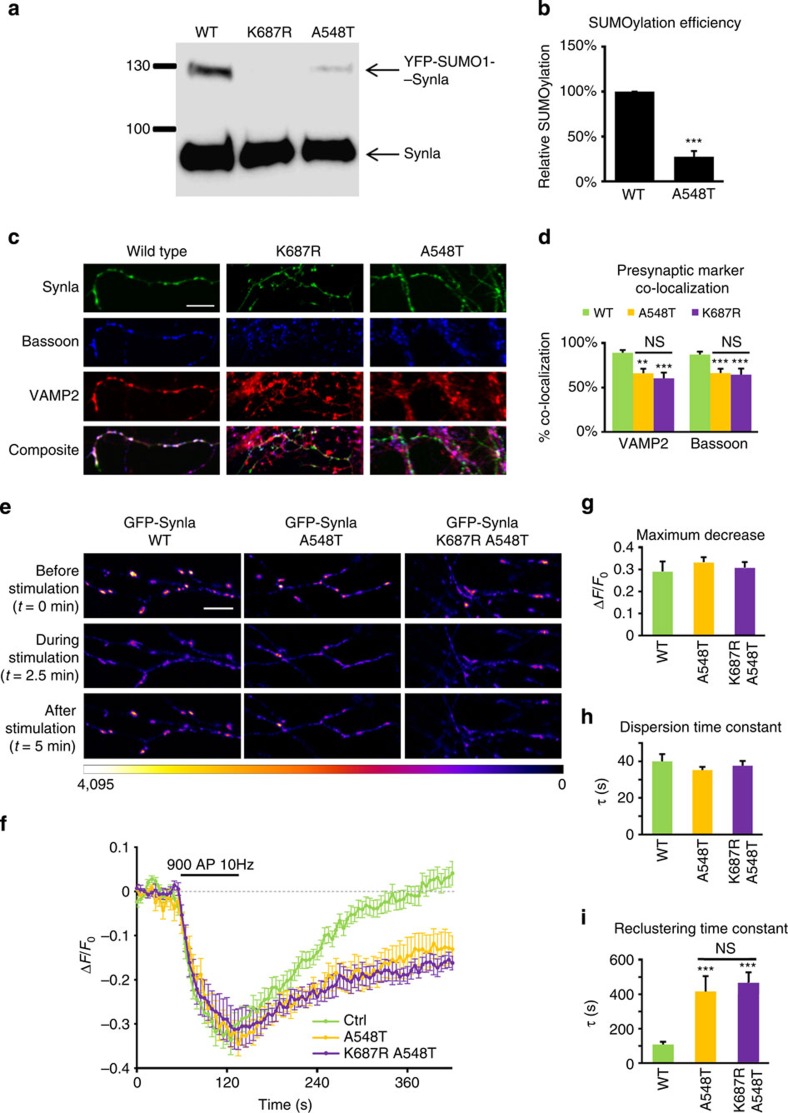
The ASD-associated A548T mutation impairs SynIa SUMOylation. (**a**) Immunoblot showing reduced SUMOylation of HA-SynIa A548T in N2A cells. (**b**) Quantitative analysis of the extent of HA-SynIa A548T SUMOylation. The blots were quantified by taking the ratio of SUMOylated band to the unmodified band normalized to HA-SynIa WT, data presented as mean ± s.e.m. **P<0.01 unpaired Student’s *t*-test, *n*=6. (**c**) Representative images showing effects of molecular replacement of endogenous SynIa with GFP-SynIa WT (*n*=13), GFP-SynIa A548T (*n*=13) and GFP-SynIa K687R (*n*=13) in hippocampal neurons. Neurons were stained with anti-bassoon as an active zone marker or anti-VAMP2 as SV marker. Scale bar, 5 μm (**d**) Quantitative analysis of synaptic co-localization as described in Fig. 4c. Data presented as mean ± s.e.m. ***P<0.001 against wild type; NS, not significant, One-way ANOVA, Bonferroni correction and *post hoc* test (**e**) Representative images of the dispersion and reclustering assay for GFP-SynIa WT, GFP-SynIa A548T and GFP-SynIa K687R-A548T. Scale bar, 5 μm. Colour scale in arbitrary fluorescence units. (**f**) Image analysis of **e** using Δ*F*/*F*_0_ plotted against time to give the dispersion and reclustering profile. Mean ± s.e.m. of GFP-SynIa WT (*n*=10), GFP-SynIa A548T (*n*=14) and GFP-SynIa K687R-A548T (*n*=13). (**g**) Histogram showing the mean ± s.e.m. of maximum dispersion at *t*=150 s. *n* numbers as in **f**. (**h**,**i**) The dispersion (*t*=60–150 s) and reclustering (*t*=150–420 s) profiles were fitted with exponential curves and time constants presented as mean ± s.e.m. ***P<0.001 against WT; NS, not significant, one-way ANOVA, *post hoc* test with Bonferroni correction. *n* numbers as in **f**.

Mutations in SynIa have been associated with epilepsy and ASD^37^. These include two nonsense mutations that abolish the E domain and other less severe mutations that do not significantly affect SV binding or phosphorylation by CamKII, but do result in defective SynIa targeting and SV exocytosis^34^. We therefore investigated the possible role of SUMOylation in the pathogenesis of these mutations. Of the mutants we investigated, the A548T mutation (analogous to A550T in human SynIa^34^) significantly decreased the levels of SynIa SUMOylation in N2A cells (Fig. 5a,b), and the defect in SV exocytosis caused by the A548T mutation is strikingly similar to that observed with K687R mutation^34^, suggesting a link between SUMOylation and ASD/epilepsy.

We next compared the presynaptic localization of GFP-SynIa WT, K687R and A548T in neurons in which endogenous SynIa was knocked down (Fig. 5c). Both non-SUMOylatable K687R and the autism mutant A548T exhibited similar SynIa targeting defects compared with WT SynIa (Fig. 5d). While we cannot formally exclude the possibility that targeting defects result from disruption of distinct pathways, these results are consistent with a common mechanism of action. To explore this in more detail, we used the SynIa dispersion assay to compare the effects of the A548T with a double A548T/K687R mutant. As expected, there were no differences in the dynamics of dispersion between the WT and either mutant, but both mutants were significantly slower in reclustering compared with WT. Moreover, the time constants for reclustering were identical for the A548T and the A548T/K687R double mutant (Fig. 5e–i) indicating that there is no additive effect of the A548T mutation in non-SUMOylatable SynIa. Again, these data are consistent with both A548T and K687R disrupting SynIa reclustering via the same pathway, and strongly suggest that the defects in SV exocytosis and SynIa targeting/clustering in this ASD mutation can be attributed to defective SUMOylation.

How the A548T mutation affects SUMOylation at the molecular level remains to be determined. Residues A548 and K687 are relatively distant from each other and *in silico* analysis of the A548T mutation does not predict any marked alteration in SynIa structure^34^. Interestingly, an *in vitro* SUMOylation assay showed no decrease in SUMOylation efficiency for A548T mutant (Supplementary Fig. 7). Since SUMOylation *in vitro* requires only Ubc9 interaction, it is unlikely that A548T mutation directly influences Ubc9 binding. Nonetheless, one possible explanation is that A548T changes SynIa conformation in such a way to occlude the SUMO consensus sequence. Alternatively, A548 may be required for SynIa interactions with a SUMO E3 ligase. Overall, our data clearly support the hypothesis that the A548T mutation-mediated defects in SynIa SUMOylation result in the dysfunction of SV dynamics. These changes, in turn, likely cause imbalances between excitatory and inhibitory stimulation that can underlie ASD and epilepsy^38^,^39^,^40^. Thus, we propose that SynIa SUMOylation plays a critical role in presynaptic function and dysfunction and that modulation of this pathway represents a potentially powerful strategy for the development of new approaches to treating synaptopathies including ASD and epilepsy.

## Conclusion

Here we show that SUMOylation of SynIa enhances its SV reclustering at the RP, which is required to maintain presynaptic responsiveness and normal synaptic transmission. We further demonstrate that the A548T mutation in SynIa reduces SUMOylation and that this may account for the epilepsy and ASD phenotype associated with this mutation. These results expand the repertoire of synaptic proteins that are regulated by SUMOylation and provide further evidence for the fundamentally important roles of SUMO modification of synaptic proteins in health and disease.

## Methods

### Molecular biology

For knockdown-rescue experiments, shRNA-resistant SynIa constructs were expressed as mCherry/GFP-IRES-SynIa cassette on a pFIV plasmid, which also expressed SynI shRNA (vector made in house, shRNA target sequence 5′-CCATGGAGAAATTGACATTAATA-3′ against SynI). For all knockdown studies, ‘Control’ cells were transfected with pFIV (expressing mCherry or GFP) without shRNA. The Flag-tagged SUMOylation vector used in bacterial SUMOylation of SynIaDE are engineered by inserting Flag tag at the N terminus of SUMO in pE1E2S1 vector. All subcloning and mutagenesis reactions were performed according to standard protocols.

### Cell culture

Embryonic cortical and hippocampal neurons were isolated from E18 Wistar *Rattus Norvergicus* embryos. Brain areas were dissected and trypsinized, before being plated on either PLL-coated 25-mm glass coverslips (for hippocampal cells) or PLL-coated six-well plates (for cortical cells). Cells were initially plated in plating media (Neurobasal media + 10% horse serum, B27 supplement, 2 mM Glutamax, 1 × penicillin/streptomycin), which after 24 h was replaced with feeding media (Neurobasal media, B27 supplement, 1.2 mM Glutamax, 1 × penicillin/streptomycin). For more details, see ref. 41. For biochemistry, cortical neurons were used at DIV18. For SypHy experiments, hippocampal neurons are transfected at DIV9-10 and imaged 5 days later. For dispersion/reclustering assay and localization experiments, hippocampal neurons are transfected at DIV9-10 and imaged 7 days later. All neuronal transfections were performed using Lipofectamine 2000 (Invitrogen) according to manufacturer’s instructions.

### Biochemistry

SUMO-1 co-immunoprecipitation was performed from whole rat brain lysate. Brain tissue (250 mg) was flash frozen and pulverized under liquid nitrogen. The resulting powder was dissolved in high SDS lysis buffer (25 mM HEPES, 500 mM NaCl, 1% Triton X-100, 2% SDS, 0.5% Sodium deoxycholate, pH 7.4) and sonicated. The lysate was subsequently diluted 10-fold with RIPA dilution buffer (25 mM HEPES, 500 mM NaCl, 1% Triton X-100, 0.5% Sodium deoxycholate, pH 7.4), which was used for pull down with 4 μg of anti-SUMO-1 D11 antibody (Santa Cruz, # sc-5308) bound to Protein A/G magnetic beads (Pierce). Western blotting was performed using anti-SynI antibody at 1:5,000 (BD biosciences, #611392).

For the N2A SUMOylation assay^42^, cells were transfected with HA-SynIa, Flag-Ubc9 and either active YFP-SUMO-1-GG or non-conjugatable YFP-SUMO-ΔGG for 48 h before lysis on ice in buffer containing 20 nM NEM. GFP-Trap (Chromotek) was used for GFP-tag pull down according to the manufacturer’s instructions. Western blotting was performed using rat anti-HA antibody at 1:1,000 (Roche, # 11583816001)

For GST-Ubc9 pull-down assay, GST-Ubc9 was expressed in BL21(DE3) *E. coli* under standard isopropylthiogalactoside induction. Harvested bacteria were lysed (lysis buffer: 25 mM HEPES, 500 mM NaCl, 2 mM DTT, 1% Triton X-100) and incubated with glutathione resin (GE Healthcare). After washing extensively, the resin was then incubated with cell lysate from cortical neuronal culture or HEK293T in lysis buffer, obtained by resuspending and sonicating cells in lysis buffer (25 mM HEPES, 150 mM NaCl, 1% Triton X-100, pH 7.4). Western blotting was performed using anti-SynI (for neuronal lysate) or anti-HA antibody (for HEK293T lysate).

Where cropping of western blots was performed, the full blots are supplied (Supplementary Fig. 8), with boxed areas indicating the cropped areas used.

### Bacterial expression and purification of SynIa constructs

Bacterial expression of His-HA-SynIaDE was carried out in BL21(DE3) *E. coli* using a standard isopropylthiogalactoside overnight induction protocol. The harvested bacteria were lysed by sonication in Nickel-Binding Buffer (25 mM HEPES, 500 mM NaCl, 20 mM Imidazole, protease inhibitors, pH 7.4). Precleared bacterial lysate was then load onto Ni-NTA resin. After extensive washes, the protein was eluted with elution buffer (25 mM HEPES, 200 mM imidazole, pH 7.4), then concentrated to 2 mg ml^−1^ before snap freezing for storage at −80 °C. SUMOylated SynIaDE was produced by co-expression of His-HA-SynIaDE and Flag-SUMOylation plasmid in BL21(DE3) *E. coli* and metal-affinity purification described above and the eluate loaded onto a Flag-affinity gel (SIGMA). Retained proteins were eluted using 50 μg ml^−1^ Flag peptide (SIGMA) in 25 mM HEPES, pH 7.4. Eluates were then snap-frozen for storage at −80 °C.

### SV-binding assay

Whole rat brain was homogenized in 10 ml ice-cold buffered sucrose (4 mM HEPES, 320 mM Sucrose, pH 7.3) using a glass-Teflon homogenizer. The homogenate was then centrifuged in a fixed angle rotor at 800*g* for 10 min. The supernatant was centrifuged at 9,200*g* for 15 min and then the subsequent supernatant was discarded. The pellet was resuspended with 10 ml of buffered sucrose and then centrifuged at 10,200*g* for 15 min. The supernatant was discarded and the pellet was resuspended in 1 ml of buffered sucrose. Ice-cold water (9 ml) was then added and the suspension was homogenized with a glass-Teflon homogenizer briefly at maximum speed. HEPES-NaOH (1 M, pH 7.4) was added to the suspension to a final concentration of 3 mM, and was incubated on ice for 30 min, then centrifuged at 25,000*g* for 20 min. The pellet, containing crude SVs, was retained. For more details, see ref. 43. SVs were resuspended in 10 ml buffered sucrose (5 mM HEPES, pH 7.4, 300 mM Sucrose) and 2.5 ml of SV suspension was mixed with either 3 μg of SUMOylated or non-SUMOylated/SENP-deSUMOylated His-HA-SynIa-DE and incubated at 4 °C with gentle agitation for 1 h. The SVs were then collected by ultracentrifugation at 55,000*g* for 2 h, resuspended in 200 μl buffered sucrose and the extent of His-HA-SynIa-DE binding was assessed by western blot analysis using an anti-HA antibody. SENP treatment of SUMO-SynIaDE was performed by adding purified recombinant SENP (catalytic domain only) at 1:100 mass ratio to SUMOylated His-HA-SynIaDE at incubate at room temperature for 30 min.

### Immunocytochemistry

Immunocytochemistry was performed following paraformaldehyde fixation of cells and permeabilization with 0.1% Triton X-100. For presynaptic co-localization assays, anti-bassoon at 1:500 (Abcam, #ab82958) and anti-VAMP2 at 1:1,000 (Synaptic Systems, #104202) was used to stain thepresynaptic terminal. The number of GFP puncta positive for bassoon or VAMP2 were counted and reported as a percentage of total GFP puncta. Each field is counted as one *n* and consists of at least 20 puncta and at least 3 independent neuronal culture preparations were assessed. For quantification in the knockdown-rescue experiments, anti-SynI at 1:500 (Novua # NB300-104) was used to assess SynI levels in mCherry-expressing neurons. All quantification was performed using ImageJ software.

### SypHy experiments

Neurons co-transfected with SynIa knockdown, or knockdown-rescue constructs (see Molecular Biology section for details) were mounted in a Warner stimulation chamber (Harvard Apparatus), connected to a Digitimer Constant Voltage stimulator and a Master-8 pulse train generator. Exocytosis was triggered using electrical field stimulation, using 1 ms pulses (APs) of 50 V with a paradigm of 600 APs at 20 Hz. All experiments were performed in HBS with 50 μM D-AP5, 25 μM CNQX and 1 μM bafilomycin A. Perfusion with NH_4_Cl (pH 7.4), was used to reveal total levels of SypHy loading at the end of each experiment. Images were taken at 0.5 Hz using a CCD camera with a GFP filter cube. For each cell, 10 regions of interest (ROIs) with area of 2.13 × 2.13 μm were manually placed covering single SypHy puncta chosen blindly. Average fluorescence signal were measured with non-responsive ROIs discounted. Data were first normalized to background levels (that is, Δ*F*/*F*_0_), and then expressed as a percentage of fluorescence after NH_4_Cl perfusion (*F*_max_) or after stimulation (*F*_post-stim_). Extrasynaptic ROIs were used to correct for background fluorescence. For more details, please see ref. 17.

### SynIa dispersion and reclustering assay

GFP-SynIa-transfected neurons were mounted in the electrical field stimulation chamber, filled with HBS supplemented with 25 μM APV and 50 μM CNQX. Confocal images were recorded at 0.2 Hz for 10 min using 488 nm argon laser excitation, with a 500–530-nm band-pass filter. Images were scanned at 512 × 512 resolution with suitable gain and offset for the best contrast and avoiding saturation of signals. This recording was to establish the effect of photobleaching, and therefore no stimulation was performed. After the recording was finished, a nearby region of the same neuron was imaged with identical settings. For this recording, 900 AP at 10 Hz (90 s) electrical stimulation (50 V, 1 ms pulses) were delivered 1 min after the start of image recording. For each cell, seven ROIs with area of 0.83 × 0.83 were manually placed covering single GFP puncta, chosen blindly at *t*=0. Average fluorescence signals were measured with non-responsive ROIs discounted. Changes in fluorescence from initial values were normalized to initial levels (that is, Δ*F*/*F*_0_). The same analysis was performed on the unstimulated recording to correct for photobleaching. To calculate the dispersion rate, the Δ*F*/*F*_0_ at *t*=60–150 s of each neuron were fitted with a single-fall exponential decay as follows




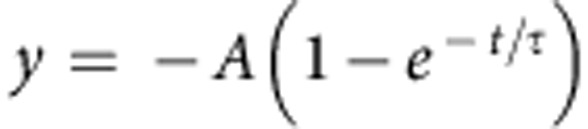




where *A* is the plateau of the curve and *τ* is the time constant that reflects the rate of increase. The *τ* from neurons transfected with the same construct were averaged for comparison between constructs with statistical testing.

To calculate the reclustering rate, the Δ*F*/*F*_0_ at *t*=150–600 s of each neuron were fitted with a single-fall exponential decay as follows




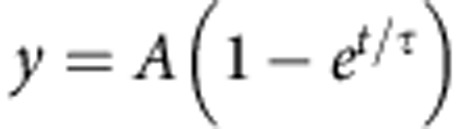




where *A* is the plateau of the curve and *τ* is the time constant that reflect the rate of increase. The *τ* from neurons transfected with the same construct were averaged and then compared between different constructs with statistical testing.

For further details, see ref. 35.

### Statistical analysis

Graphpad Prism software (Graphpad Inc.) was used for statistical analysis. Data were analysed using one-way analysis of variance (ANOVA) with Bonferroni’s *post hoc* test (for comparisons between multiple data sets) or two-tailed Student’s *t*-test (comparison between two data sets). Curve fitting in Figs 2, 4 and 5 was performed using Graphpad Prism, fitting to a single exponential rise function *y*=*a*(1−*e*^−*t*/τ^) where *τ* is the time constant.

### Sequence alignment

Sequence alignment is done in UGENE using ClustalX algorithm. Synapsins sequences were obtained from NCBI.

## Additional information

**How to cite this article:** Tang, L. T.-H. *et al*. SUMOylation of Synapsin Ia maintains synaptic vesicle availability and is reduced in an autism mutation. *Nat. Commun.* 6:7728 doi: 10.1038/ncomms8728 (2015).

## Supplementary Material

Supplementary Information Supplementary Figure 1-8

## References

[b1] Rizzoli S. O. & Betz W. J. Synaptic vesicle pools. Nat. Rev. Neurosci. 6, 57–69 (2005). 15611727 10.1038/nrn1583

[b2] Ceccaldi P. E. . Dephosphorylated synapsin I anchors synaptic vesicles to actin cytoskeleton: an analysis by videomicroscopy. J. Cell Biol. 128, 905–912 (1995). 7876313 10.1083/jcb.128.5.905 PMC2120389

[b3] Cesca F., Baldelli P., Valtorta F. & Benfenati F. The synapsins: key actors of synapse function and plasticity. Prog. Neurobiol. 91, 313–348 (2010). 20438797 10.1016/j.pneurobio.2010.04.006

[b4] Bykhovskaia M. Synapsin regulation of vesicle organization and functional pools. Semin. Cell Dev. Biol. 22, 387–392 (2011). 21827866 10.1016/j.semcdb.2011.07.003

[b5] Verstegen A. M. . Phosphorylation of synapsin I by cyclin-dependent kinase-5 sets the ratio between the resting and recycling pools of synaptic vesicles at hippocampal synapses. J. Neurosci. 34, 7266–7280 (2014). 24849359 10.1523/JNEUROSCI.3973-13.2014 PMC6608192

[b6] Jovanovic J. N. . Opposing changes in phosphorylation of specific sites in synapsin I during Ca2+-dependent glutamate release in isolated nerve terminals. J. Neurosci. 21, 7944–7953 (2001). 11588168 10.1523/JNEUROSCI.21-20-07944.2001 PMC6763853

[b7] Wilkinson K. A., Nakamura Y. & Henley J. M. Targets and consequences of protein SUMOylation in neurons. Brain Res. Rev. 64, 195–212 (2010). 20382182 10.1016/j.brainresrev.2010.04.002 PMC3310160

[b8] Craig T. J. & Henley J. M. Protein SUMOylation in spine structure and function. Curr. Opin. Neurobiol. 22, 480–487 (2012). 22054923 10.1016/j.conb.2011.10.017 PMC3379963

[b9] Luo J. . Receptor trafficking and the regulation of synaptic plasticity by SUMO. Neuromol. Med. 15, 692–706 (2013). 10.1007/s12017-013-8253-y 23934328

[b10] Feligioni M., Nishimune A. & Henley J. M. Protein SUMOylation modulates calcium influx and glutamate release from presynaptic terminals. Eur. J. Neurosci. 29, 1348–1356 (2009). 19344328 10.1111/j.1460-9568.2009.06692.x PMC3309032

[b11] Guo C. & Henley J. Wrestling with stress: roles of protein SUMOylation and deSUMOylation in cell stress response. IUBMB Life 66, 71–77 (2014). 24470405 10.1002/iub.1244

[b12] Girach F., Craig T. J., Rocca D. L. & Henley J. M. RIM1alpha SUMOylation is required for fast synaptic vesicle exocytosis. Cell Rep. 5, 1294–1301 (2013). 24290762 10.1016/j.celrep.2013.10.039 PMC3898736

[b13] Hilfiker S. . Two sites of action for synapsin domain E in regulating neurotransmitter release. Nat. Neurosci. 1, 29–35 (1998). 10195105 10.1038/229

[b14] Monaldi I. . The highly conserved synapsin domain E mediates synapsin dimerization and phospholipid vesicle clustering. Biochem. J. 426, 55–64 (2010). 19922412 10.1042/BJ20090762

[b15] Sudhof T. C. . Synapsins: mosaics of shared and individual domains in a family of synaptic vesicle phosphoproteins. Science 245, 1474–1480 (1989). 2506642 10.1126/science.2506642

[b16] Candiani S. . The synapsin gene family in basal chordates: evolutionary perspectives in metazoans. BMC Evol. Biol. 10, 32 (2010). 20113475 10.1186/1471-2148-10-32 PMC2825198

[b17] Burrone J., Li Z. & Murthy V. N. Studying vesicle cycling in presynaptic terminals using the genetically encoded probe synaptopHluorin. Nat. Protoc. 1, 2970–2978 (2006). 17406557 10.1038/nprot.2006.449

[b18] Ikeda K. & Bekkers J. M. Counting the number of releasable synaptic vesicles in a presynaptic terminal. Proc. Natl Acad. Sci USA 106, 2945–2950 (2009). 19202060 10.1073/pnas.0811017106 PMC2650301

[b19] Li L. . Impairment of synaptic vesicle clustering and of synaptic transmission, and increased seizure propensity, in synapsin I-deficient mice. Proc. Natl Acad. Sci. USA 92, 9235–9239 (1995). 7568108 10.1073/pnas.92.20.9235 PMC40959

[b20] Rosahl T. W. . Essential functions of synapsins I and II in synaptic vesicle regulation [see comments]. Nature 375, 488–493 (1995). 7777057 10.1038/375488a0

[b21] Ryan T. A., Li L., Chin L. S., Greengard P. & Smith S. J. Synaptic vesicle recycling in synapsin I knock-out mice. J. Cell Biol. 134, 1219–1227 (1996). 8794863 10.1083/jcb.134.5.1219 PMC2120974

[b22] Buchner A. . Sumoylation of p35 modulates p35/cyclin-dependent kinase (Cdk) 5 complex activity. Neuromol. Med. 17, 12–23 (2015). 10.1007/s12017-014-8336-4 25391294

[b23] Geerts C. J., Jacobsen L., van de Bospoort R., Verhage M. & Groffen A. J. Tomosyn interacts with the SUMO E3 ligase PIASgamma. PLoS ONE 9, e91697 (2014). 24614299 10.1371/journal.pone.0091697 PMC3948876

[b24] Uchimura Y., Nakamura M., Sugasawa K., Nakao M. & Saitoh H. Overproduction of eukaryotic SUMO-1- and SUMO-2-conjugated proteins in *Escherichia coli*. Anal. Biochem. 331, 204–206 (2004). 15246018 10.1016/j.ab.2004.04.034

[b25] Wilkinson K. A., Nishimune A. & Henley J. M. Analysis of SUMO-1 modification of neuronal proteins containing consensus SUMOylation motifs. Neurosci. Lett. 436, 239–244 (2008). 18400391 10.1016/j.neulet.2008.03.029 PMC3310158

[b26] Benfenati F., Bahler M., Jahn R. & Greengard P. Interactions of synapsin I with small synaptic vesicles: distinct sites in synapsin I bind to vesicle phospholipids and vesicle proteins. J. Cell Biol. 108, 1863–1872 (1989). 2497106 10.1083/jcb.108.5.1863 PMC2115532

[b27] Benfenati F., Valtorta F., Bahler M. & Greengard P. Synapsin I, a neuron-specific phosphoprotein interacting with small synaptic vesicles and F-actin. Cell Biol. Int. Rep. 13, 1007–1021 (1989). 2517594 10.1016/0309-1651(89)90016-7

[b28] Gareau J. R. & Lima C. D. The SUMO pathway: emerging mechanisms that shape specificity, conjugation and recognition. Nat. Rev. Mol. Cell Biol. 11, 861–871 (2011). 10.1038/nrm3011 PMC3079294 21102611

[b29] Kerscher O. SUMO junction-what's your function? New insights through SUMO-interacting motifs. EMBO Rep. 8, 550–555 (2007). 17545995 10.1038/sj.embor.7400980 PMC2002525

[b30] Benfenati F., Neyroz P., Bahler M., Masotti L. & Greengard P. Time-resolved fluorescence study of the neuron-specific phosphoprotein synapsin I. Evidence for phosphorylation-dependent conformational changes. J. Biol. Chem. 265, 12584–12595 (1990). 2115521

[b31] Gitler D. . Different presynaptic roles of synapsins at excitatory and inhibitory synapses. J. Neurosci. 24, 11368–11380 (2004). 15601943 10.1523/JNEUROSCI.3795-04.2004 PMC6730366

[b32] Tang Y. . Fast vesicle transport is required for the slow axonal transport of synapsin. J. Neurosci. 33, 15362–15375 (2013). 24068803 10.1523/JNEUROSCI.1148-13.2013 PMC3782618

[b33] Easley-Neal C., Fierro J. Jr., Buchanan J. & Washbourne P. Late recruitment of synapsin to nascent synapses is regulated by Cdk5. Cell Rep. 3, 1199–1212 (2013). 23602570 10.1016/j.celrep.2013.03.031 PMC3742072

[b34] Fassio A. . SYN1 loss-of-function mutations in autism and partial epilepsy cause impaired synaptic function. Hum. Mol. Genet. 20, 2297–2307 (2011). 21441247 10.1093/hmg/ddr122

[b35] Chi P., Greengard P. & Ryan T. A. Synapsin dispersion and reclustering during synaptic activity. Nat. Neurosci. 4, 1187–1193 (2001). 11685225 10.1038/nn756

[b36] Wilkinson K. A. & Henley J. M. Mechanisms, regulation and consequences of protein SUMOylation. Biochem. J. 428, 133–145 (2010). 20462400 10.1042/BJ20100158 PMC3310159

[b37] Garcia C. C. . Identification of a mutation in synapsin I, a synaptic vesicle protein, in a family with epilepsy. J. Med. Genet. 41, 183–186 (2004). 14985377 10.1136/jmg.2003.013680 PMC1735688

[b38] Etholm L. & Heggelund P. Seizure elements and seizure element transitions during tonic-clonic seizure activity in the synapsin I/II double knockout mouse: a neuroethological description. Epilepsy Behav. 14, 582–590 (2009). 19236947 10.1016/j.yebeh.2009.02.021

[b39] Greco B. . Autism-related behavioral abnormalities in synapsin knockout mice. Behav. Brain Res. 251, 65–74 (2013). 23280234 10.1016/j.bbr.2012.12.015 PMC3730181

[b40] Lignani G. . Epileptogenic Q555X SYN1 mutant triggers imbalances in release dynamics and short-term plasticity. Hum. Mol. Genet. 22, 2186–2199 (2013). 23406870 10.1093/hmg/ddt071 PMC3652419

[b41] Martin S. & Henley J. M. Activity-dependent endocytic sorting of kainate receptors to recycling or degradation pathways. EMBO J. 23, 4749–4759 (2004). 15549132 10.1038/sj.emboj.7600483 PMC535095

[b42] Craig T. J. & Henley J. M. SUMOylation, Arc and the regulation homeostatic synaptic scaling: implications in health and disease. Commun. Integr. Biol. 5, 634–636 (2012). 23739045 10.4161/cib.21712 PMC3541335

[b43] Huttner W. B., Schiebler W., Greengard P. & De Camilli P. Synapsin I (protein I), a nerve terminal-specific phosphoprotein. III. Its association with synaptic vesicles studied in a highly purified synaptic vesicle preparation. J. Cell Biol. 96, 1374–1388 (1983). 6404912 10.1083/jcb.96.5.1374 PMC2112660

[b44] Uchimura Y., Nakao M. & Saitoh H. Generation of SUMO-1 modified proteins in E. coli: towards understanding the biochemistry/structural biology of the SUMO-1 pathway. FEBS Lett. 564, 85–90 (2004). 15094046 10.1016/S0014-5793(04)00321-7

