# Supplementary material for: SUMOylation of synapsin Ia maintains synaptic vesicle availability and is reduced in an autism mutation
Source: Nat Commun. 2015 Jul 15;6:7728. doi: 10.1038/ncomms8728 (PMC4504226; doi:10.1038/ncomms8728)
Supplement: Supplementary Information — Supplementary Figure 1-8 [file ncomms8728-s1.pdf]

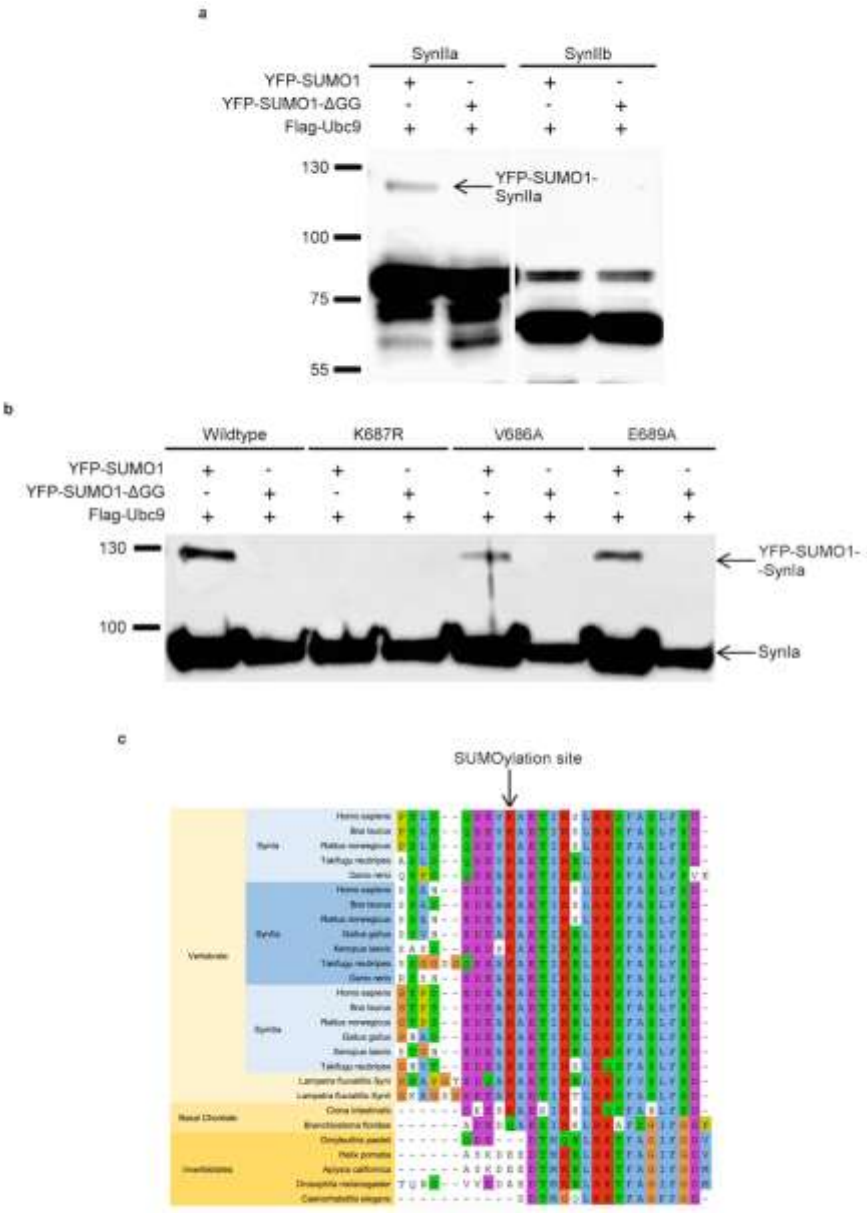

Supplementary Figure 1

Possible SUMOylation of other Synapsin isoforms

a) Representative immunoblot showing that SynIIa, but not SynIIb, is robustly SUMOylated in N2A SUMOylation assay.
b) Representative immunoblot showing that SynIa V686A and E689A mutant can be SUMOylated at a lower level in N2A SUMOylation assay.
Immunoblots were obtained through anti-HA western blot of N2A lysate described in figure 1a.
c) Sequence alignment of the Synapsin E domains generated in UGENE using ClustalX algorithm and colour coded to highlight the conserved SUMOylation site in vertebrates.

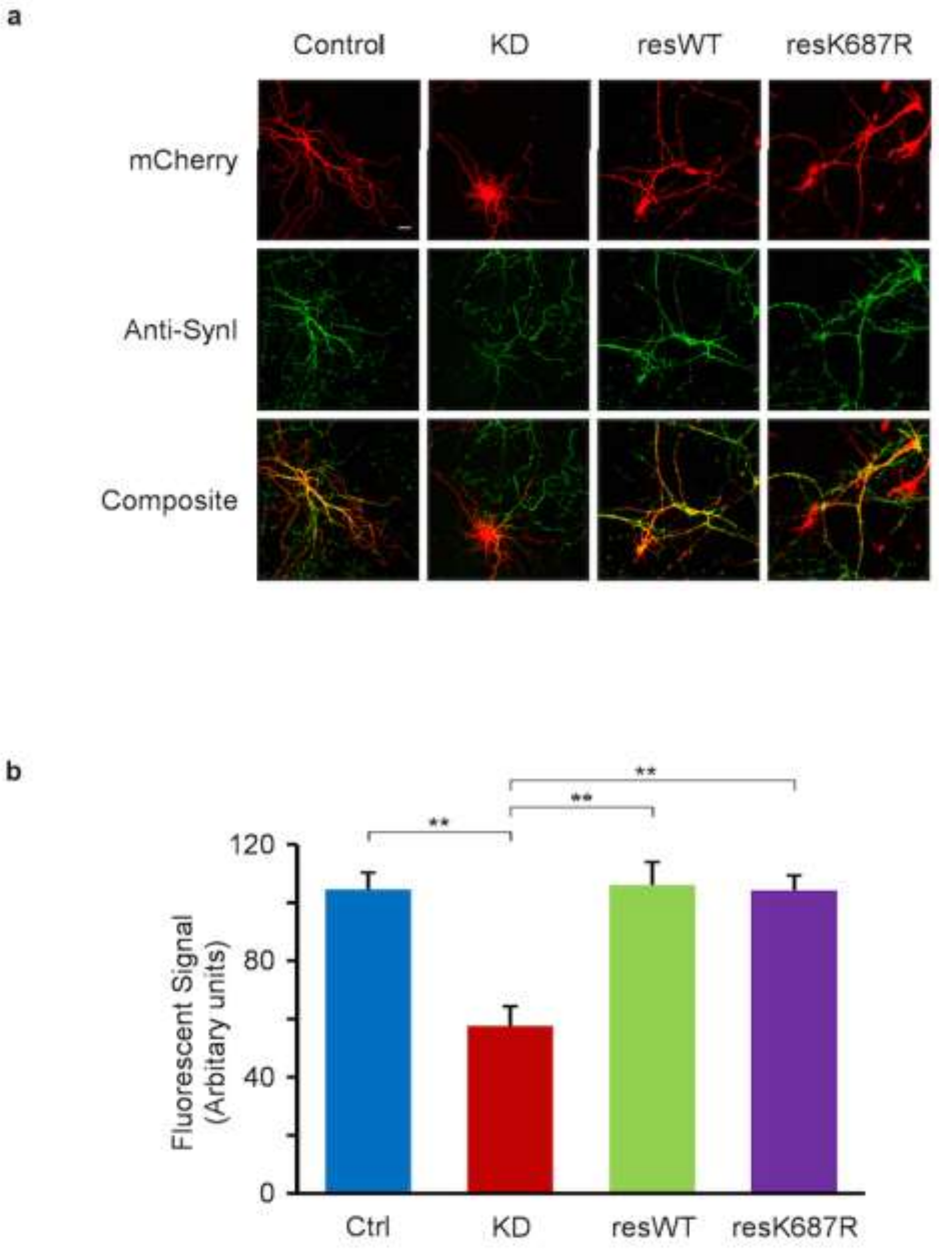

Supplementary Figure 2

SynI expression levels in knockdown and molecular replacement experiments.

a) Representative images of hippocampal neurons transfected with Control (n=9), SynIa knockdown (KD) (n=10), KD-rescue with GFP-SynIa WT (n=9) or KD-rescue with GFP-SynIa K687R (n=9). Cells were fixed and immunostained with anti-SynI antibody (with Cy2 secondary). Scale bar = 5 µm.

b) Quantification of the level of anti-SynI antibody immunofluorescence in mCherry positive neurons. Quantification was performed using the mCherry signal to delineate region of interest. The average level of Cy2 signal within the ROI was then measured. All Cy2 signals are imaged with the same settings. The data are presented as mean ± SEM. ** $p<0.01$, one-way ANOVA, post-hoc test with Bonferroni correction. n numbers as in (a).

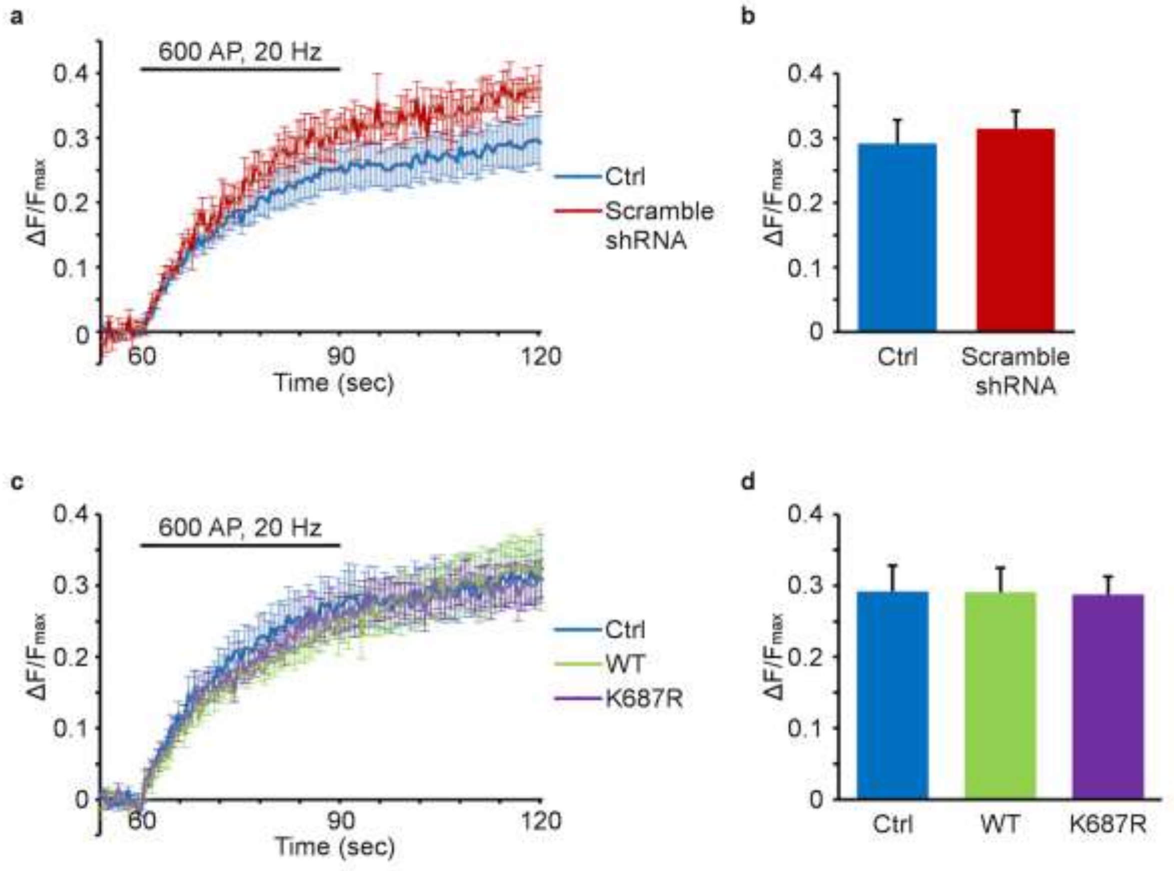

Supplementary Figure 3

Use of scramble shRNA or expression of SynIa WT or K687R under non-knockdown background does not change the overall exocytosis.

a) SypHy exocytosis assay on mCherry Control (Ctrl, n=8) and scrambled shRNA mCherry control (Scramble shRNA, n=7). SypHy fluorescence data were normalized to the maximum SypHy signal obtained after NH_4Cl application ($\Delta F/F_{max}$) and plotted as the mean ± SEM.

b) Quantification of data shown in **a)** plotted as the mean ± SEM of the average $\Delta F/F_{max}$ t = 90 – 100. No significant differences were observed, unpaired Student's t-test

c) SypHy exocytosis assay on mCherry Control (Ctrl, n=8), SynIa wildtype overexpression (WT, n=8) and SynIa K687R overexpression (K687R, n=10). SypHy fluorescence data were normalized to the maximum SypHy signal obtained after NH_4Cl application ($\Delta F/F_{max}$) and plotted as the mean ± SEM.

d) Quantification of data shown in **c)** plotted as the mean ± SEM of the average $\Delta F/F_{max}$ t = 90 – 100. No significant differences were observed, one-way ANOVA.

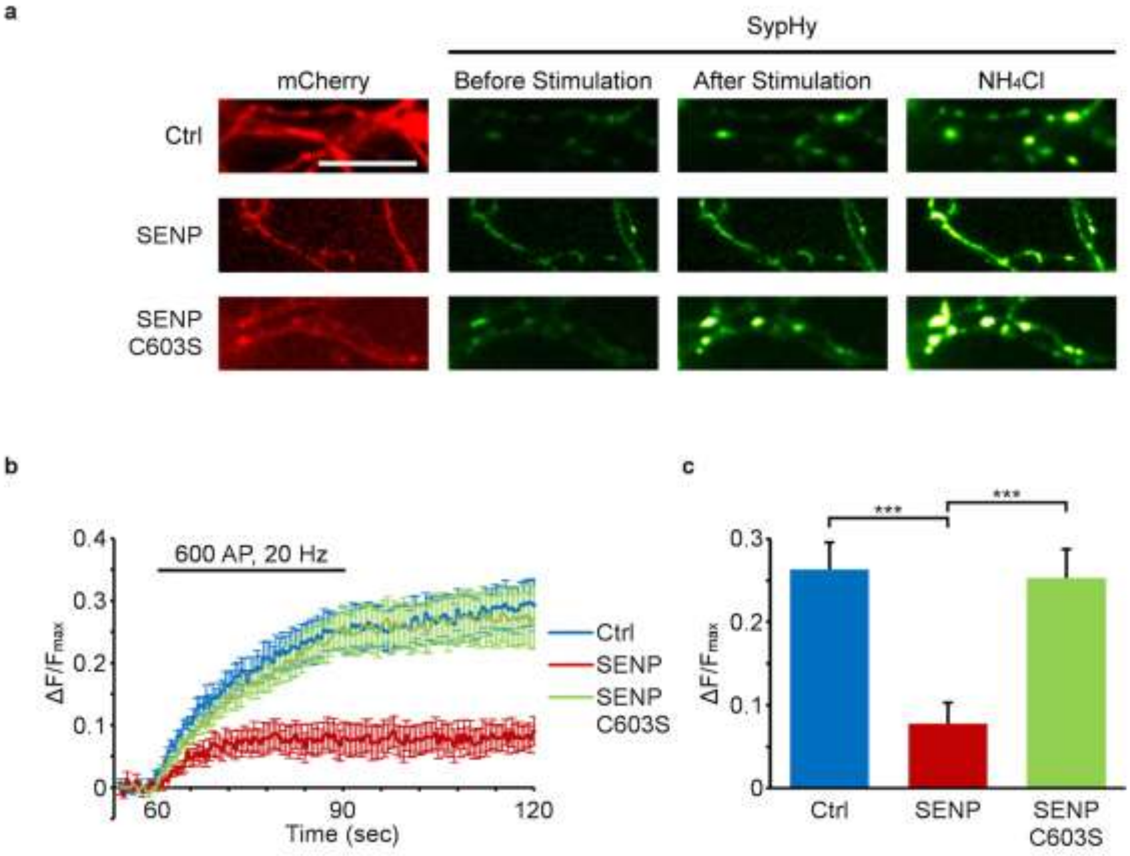

Supplementary Figure 4

SENP expression in hippocampal neuron decreases overall SV exocytosis

a) Representative images showing SynIa knockdown and molecular replacement in DIV 12 hippocampal neuron and imaged live 3 days later. SypHy protocol was identical to figure 2a. Images are representative of the SypHy signal before and after stimulation (t = 0 sec and 90 sec respectively), and after NH_4Cl profusion (t = 180 sec). mCherry Control (Ctrl, n=8), SENP expression (n=8), and inactive SENP C603S (n=8) . Scale bar = 10 µm

b) SypHy fluorescence data were normalized to the maximum SypHy signal obtained after NH_4Cl application ($\Delta F/F_{max}$) and plotted as the mean ± SEM.

c) Quantification of data shown in **b)** plotted as the mean ± SEM of the average $\Delta F/F_{max}$ t = 90 - 100 sec***: p<0.001, one-way ANOVA, Post-hoc test with Bonferroni correction.

Supplementary Figure-5 (Tang, Craig and Henley)

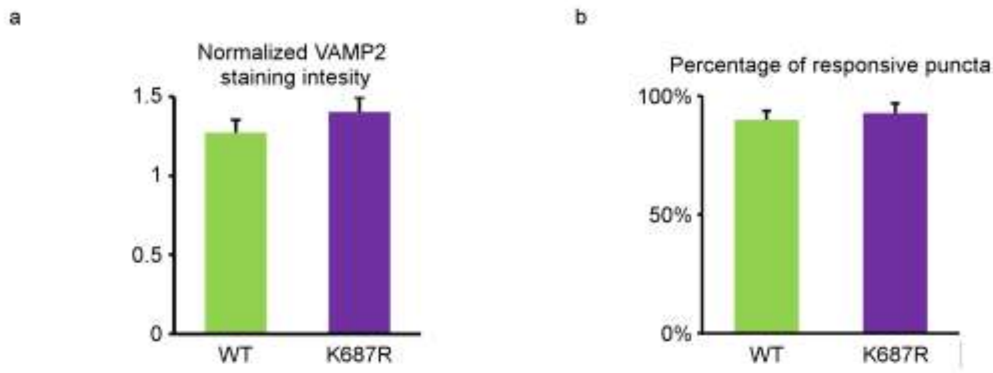

Supplementary Figure 5

a) No difference in staining intensity of SV protein (VAMP2) between GFP-SynIa (n=14 cells) and GFP-SynIa K6987R (n=15 cells) positive puncta, normalized against intensity of corresponding bassoon staining to account for variation in size of presynaptic terminal. Data are the mean ± SEM. Unpaired Student's t-test. This suggests that the K687R mutant does not alter SV total size.

b) No difference between number of responsive puncta between GFP SynIa-WT (n=9) or K687R (n=12) in the dispersion and re-clustering assay. Data plotted as the mean ± SEM. Number of responsive puncta were counted and normalized against total number of puncta. No significance differences were observed, unpaired Student's t-test. This suggests that under non-knockdown background, K687R mutant have similar association to SVs as wildtype SynIa.

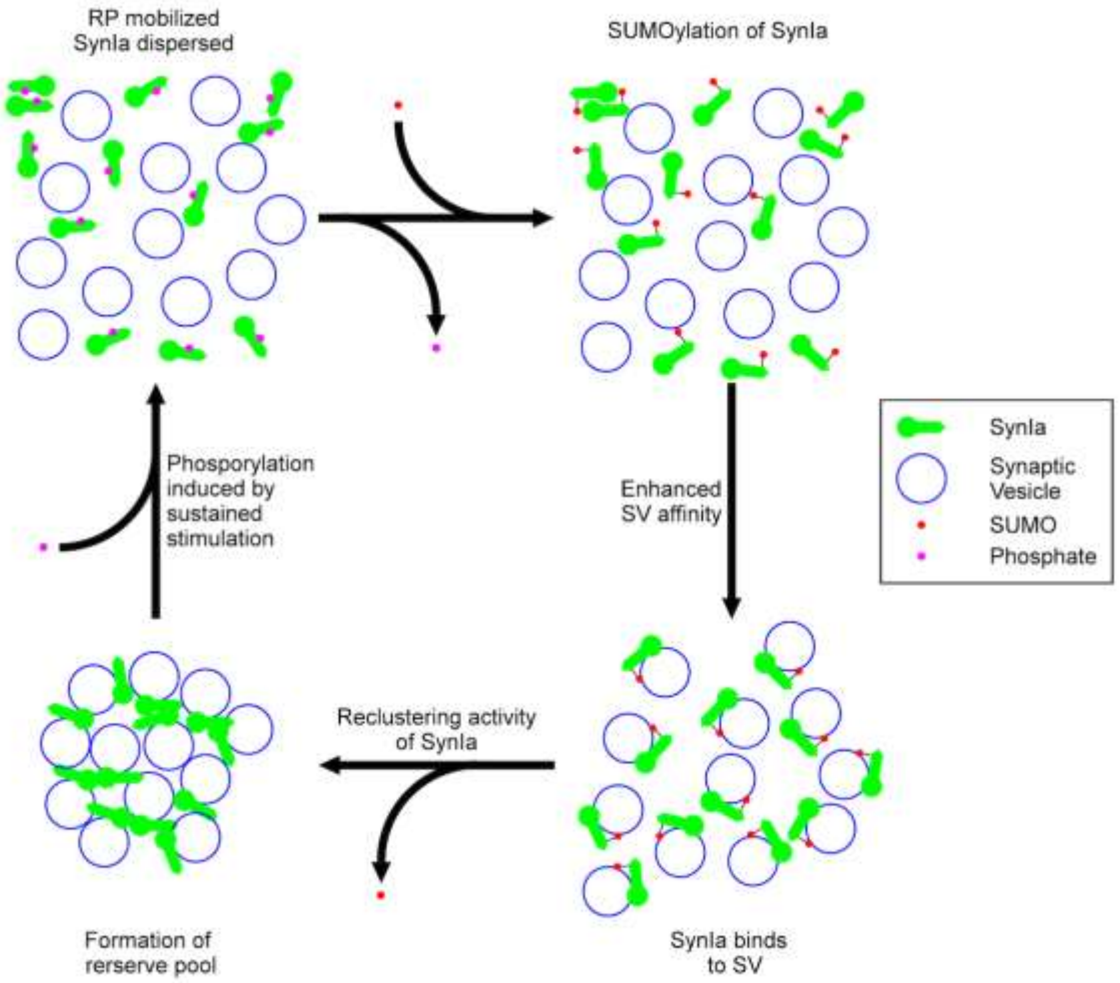

Supplementary Figure 6

Graphical representation of the hypothesized functional mechanism of SynIa SUMOylation During sustained stimulation, SynIa dissociats from SVs due to phosphorylation by CamKII and PKA. SynIa is subsequently dephosphorylated and SUMOylated. SUMOylation enhances SynIa SV affinity, likely due to additional protein interactions of SUMO with SV proteins. SV-SynIa binding remains stable after SUMO deconjugation. SV bound with SynIa are then able to crosslink and anchor to the actin cytoskeleton, clustering to reform the recycling pool.

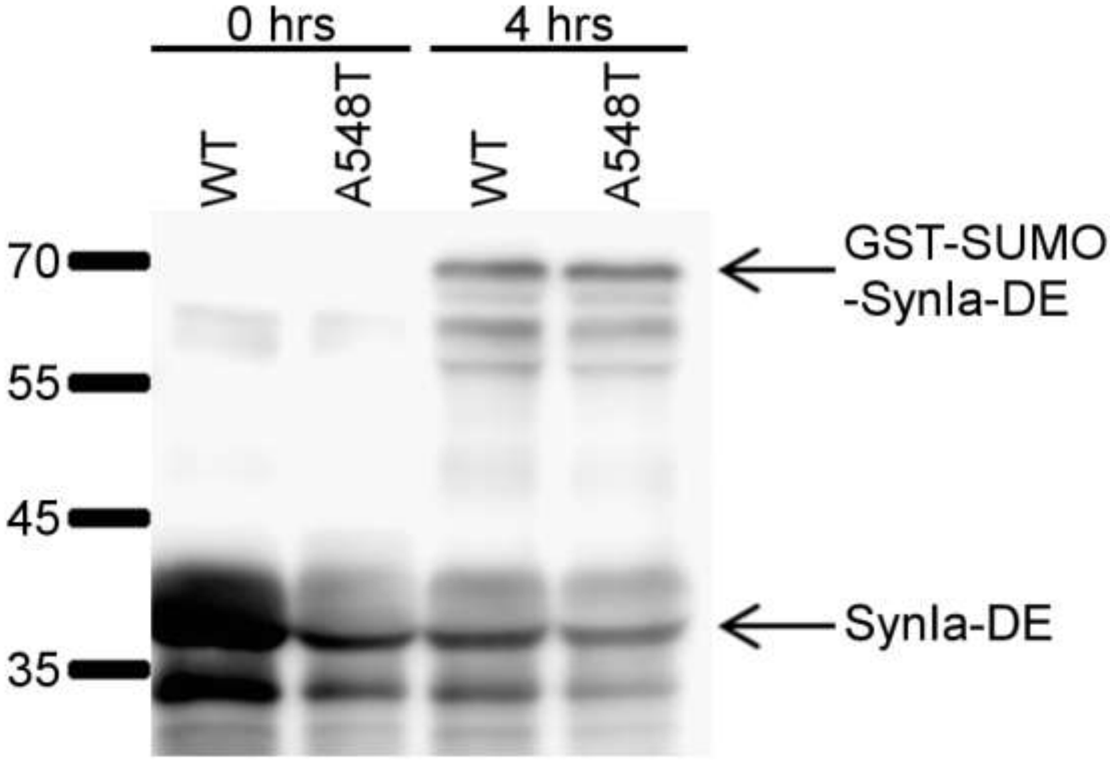

Supplementary Figure 7

No difference observed in *in vitro* SUMOylation assay between wildtype and A548T SynIaDE. GST-SUMO, His-Ubc9, SAE1/2, SynIaDE and SynIaDE A548T mutant are bacterially expressed and purified. SynIaDE, GST-SUMO1, His-Ubc9 and GST-SAE1/2 were then mixed in molar ratio of 1 : 2 : 1 : 0.01, in 25 mM Tris-HCl, 150 mM NaCl, 2 mM DTT, 10 mM $MgCl_2$, 5 mM ATP, pH 7.5. The reactions were incubated at 37°C for 4 hours, and analysed with anti-SynI western blot.

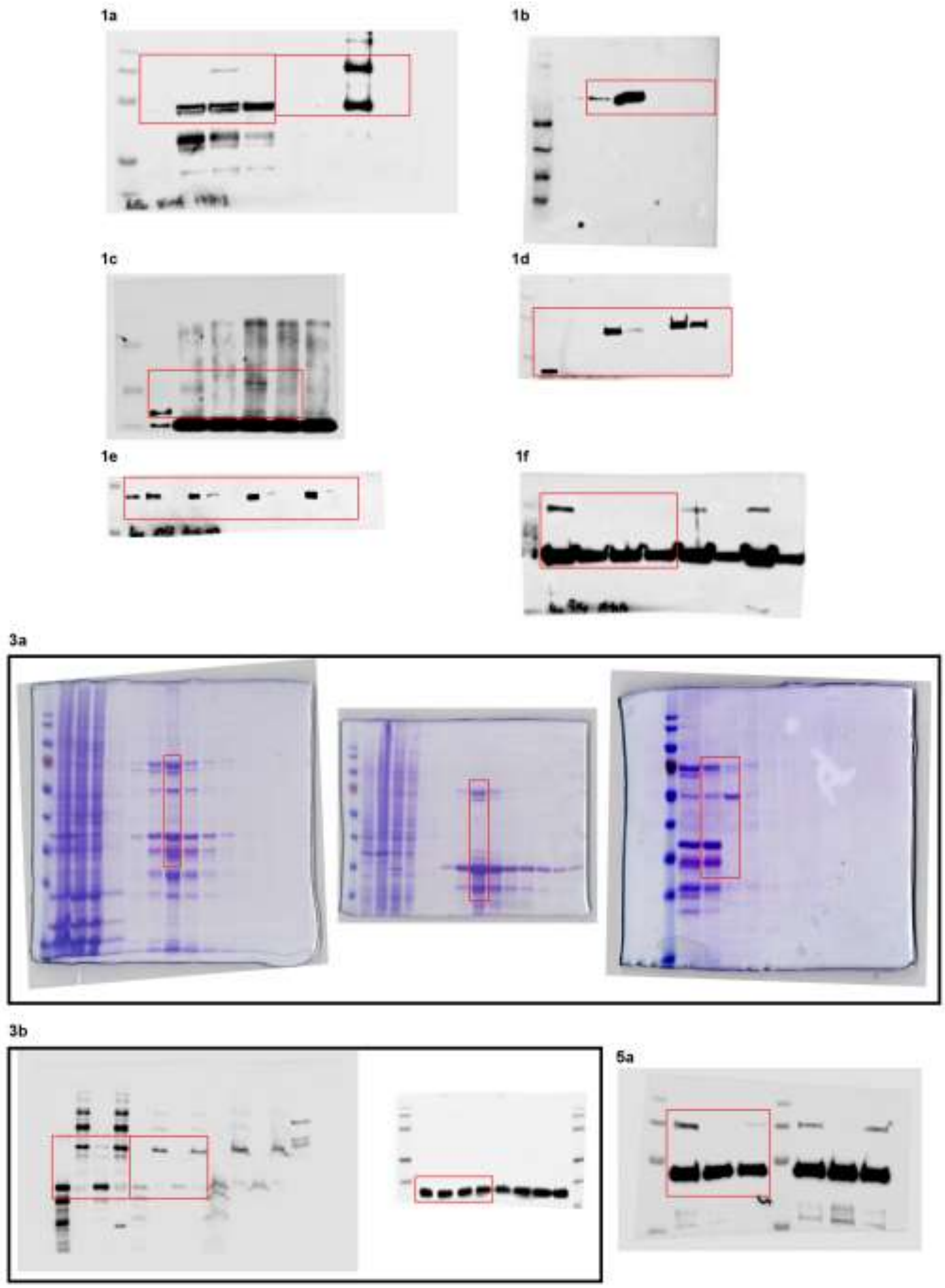

Supplementary Figure 8

Original (uncropped) blots and gels.

Cropped areas used are indicated with red boxes.
